# Construction and internal cohort verification of clinical-imaging-based nomogram for early diagnosis in Takayasu arteritis

**DOI:** 10.3389/fmed.2026.1743349

**Published:** 2026-02-03

**Authors:** Jing Chen, Hongsheng Sun

**Affiliations:** 1 Department of Rheumatology and Immunology, Shandong Provincial Hospital Affiliated to Shandong First Medical University, Jinan, China; 2 Department of Rheumatology and Immunology, Shandong Provincial Hospital, Cheeloo College of Medicine, Shandong University, Jinan, China

**Keywords:** early diagnosis, nomogram, radiomics, Takayasu arteritis, computed tomography

## Abstract

**Objective:**

Takayasu arteritis (TAK) is a chronic large-vessel vasculitis. This study aimed to develop and internally validate a nomogram model integrating clinical indicators, conventional imaging features, and radiomics features for the early diagnosis of TAK.

**Methods:**

A total of 356 patients suspected of having TAK in our hospital were retrospectively included. They were randomly divided into a training set (*n* = 249) and a validation set (*n* = 107) in a ratio of 7:3. In the training set, Lasso regression was used to screen the influencing factors associated with TAK, and a Nomogram prediction model was constructed. The predictive efficacy and clinical application value of the model were evaluated by receiver operating characteristic (ROC) curve, calibration curve, and decision curve analysis (DCA).

**Results:**

In the training set, 75 cases (30.12%) were diagnosed with early-stage TAK, and in the validation set, 32 cases (29.91%) were diagnosed. There were no statistically significant differences in the incidence of TAK and clinical characteristics between the two groups (*p* > 0.05). In the training set, multivariate logistic regression identified the following independent predictors for early-stage TAK: intermittent claudication of the limbs, vascular murmur, erythrocyte sedimentation rate (ESR), C-reactive protein (CRP), the thickest part of the vascular wall, degree of vascular wall enhancement, and contrast were identified as risk factors (all OR > 1), while uniformity and energy was identified as a protective factor (OR < 1) (all *p* < 0.05). The C-index was 0.767 and 0.733, respectively. The mean absolute errors of the agreement between the predicted and actual values were 0.163 and 0.180, respectively. The results of the Hosmer-Lemeshow test were χ^2^ = 7.937, *p* = 0.440 and χ^2^ = 11.924, *p* = 0.155, respectively. The ROC curve showed that the areas under the curve (AUC) of the nomogram model for predicting whether patients were diagnosed with TAK in the early-stage diagnosis in the training set and validation set were 0.767 (95% CI: 0.684–0.850) and 0.733 (95% CI: 0.616–0.849) respectively, with sensitivities and specificities of 0.847, 0.660 and 0.720, 0.500, respectively.

**Conclusion:**

This study successfully constructed and validated a comprehensive nomogram model, which can provide individualized and non-invasive risk assessment for the early diagnosis of TAK and contribute to clinical decision-making.

## Introduction

Takayasu’s Arteritis (TAK) is a chronic granulomatous vasculitis of unknown etiology (1). It mainly involves the aorta and its major branches, predominantly affects young women, and often leads to vascular stenosis, occlusion, dilation, or aneurysm formation, with a relatively high disability rate (2). The early-stage symptoms of TAK are non-specific, such as fever, fatigue, and joint pain, resulting in an average diagnostic delay of months or even years, which poses a serious threat to the long-term quality of life of patients (3).

Currently, the diagnosis of TAK mainly relies on the comprehensive evaluation of clinical manifestations, laboratory tests, and imaging examinations. Although the classification criteria proposed by the American College of Rheumatology in 1990 are widely used, they are mainly applicable to typical late-stage cases and have limited diagnostic sensitivity for early-stage patients (4). In terms of laboratory tests, erythrocyte sedimentation rate and C-reactive protein are important indicators reflecting disease activity, but their specificity is not high, and they can also be elevated in many infectious, neoplastic, and other autoimmune diseases. In addition, approximately one-third of TAK patients may have normal inflammatory indicators at the time of consultation, which further increases the difficulty of early diagnosis.

Although computed Tomography Angiography (CTA) has become an important non-invasive diagnostic tool due to its high spatial resolution and ability to clearly show characteristic changes such as vascular wall thickening and enhancement (5), for early or atypical cases, conventional imaging diagnosis still highly depends on the experience of physicians and is subjective. Radiomics, as an emerging technology, can extract and analyze deep-level features in medical images that are unrecognizable to the human eye through high-throughput methods, converting imaging data into high-value quantitative data, and has shown great potential in oncology and other fields (6). It is hypothesized that the microstructural changes in the diseased vascular wall of TAK can be captured by radiomics even when the macroscopic morphological changes are not obvious, thus providing new clues for early diagnosis.

A nomogram, as an intuitive statistical prediction tool, can convert complex regression equations into an easy-to-use visual scoring system, enabling clinicians to quickly calculate the disease risk probability of individual patients. In recent years, nomograms have been successfully applied in the individualized diagnosis and treatment of various diseases. However, there is currently a lack of a nomogram model integrating clinical features, conventional imaging markers, and radiomics features for the early diagnosis of TAK.

Based on the above background, this study aims to develop and internally validate a comprehensive clinical-radiomics nomogram model by systematically collecting the complete data of clinically suspected TAK patients. We hypothesize that a prediction model established by integrating multi-source information can significantly improve the accuracy of early diagnosis of TAK, provide a reliable risk assessment tool for clinicians, and ultimately achieve the goal of early intervention and improved patient prognosis.

## Materials and methods

### Study materials

This study is a single-center, retrospective observational cohort study. The research protocol was approved by the ethics committee of our hospital, and informed consent from patients was waived. Retrospectively, patients suspected of having TAK who visited our hospital from January 2018 to December 2024 were collected. Inclusion criteria: (1) Completion of CTA examination of the aorta and its branches (7), (2) Availability of complete clinical and laboratory data. Exclusion criteria: (1) Poor image quality, (2) Diagnosis of other definite vascular diseases (such as atherosclerosis, arterial dissection) or late-stage TAK with obvious vascular stenosis, occlusion, dilation, or aneurysm formation, (3) History of vascular surgery or interventional treatment.

Patients were considered ‘suspected of having TAK’ if they met the following criteria at initial consultation: (1) Presented with non-specific systemic symptoms including unexplained fever (duration >1 week), persistent fatigue, unexplained weight loss (>5% of body weight within 3 months), or joint pain; or (2) Preliminary imaging examinations (ultrasound or plain CT) showed vascular wall thickening (aortic wall thickness >3 mm) without a clear cause. All suspected patients had no definite alternative diagnosis at the time of enrollment.

### Data collection

Baseline data of patients were collected, including demographic data, clinical symptoms, signs, and laboratory tests. Two radiologists unaware of the grouping analyzed the conventional CTA images and evaluated features such as vascular wall thickening and enhancement. The selection of clinical predictors was based on two principles: (1) Consistency with the 1990 American College of Rheumatology (ACR) classification criteria for TAK to ensure compatibility with clinical practice; (2) High incidence in preliminary screening (symptoms with incidence >20% in suspected TAK patients). Symptoms such as hypertension and BP inequality were excluded due to low incidence (<15%) in the preliminary cohort, which may lead to insufficient statistical power. All selected predictors were reviewed and confirmed by two senior rheumatologists to ensure clinical relevance.

### Imaging analysis

#### Image segmentation and feature extraction

CTA scans were performed using a 64-slice spiral CT scanner (Siemens Somatom Definition Flash) with the following parameters: tube voltage 120 kV, tube current 200–300 mAs, slice thickness 1 mm, reconstruction interval 0.5 mm. Contrast agent (iohexol, 300 mgI/mL) was injected via the antecubital vein at a rate of 3.0–3.5 mL/s, with a total dose of 1.5 mL/kg body weight. Arterial-phase images were acquired 25–30 s after the start of contrast injection using the bolus tracking technique. Using ITK-SNAP software (version 4.13.0), a three-dimensional region of interest (3D-ROI) was manually segmented along the aortic wall (from the aortic root to the abdominal aorta above the renal arteries) on arterial-phase images, avoiding luminal blood flow, adjacent soft tissues, and artifacts. The thickest part of the vascular wall was identified by visual inspection of axial, coronal, and sagittal reconstructions, and measured at the maximal thickness point using the built-in caliper tool. The degree of vascular wall enhancement was graded semi-quantitatively as “strong” (enhancement intensity > 50 HU compared to pre-contrast images) or “weak” (enhancement intensity ≤ 50 HU) based on the difference in CT values between arterial-phase and pre-contrast images. Radiomics features, including first-order statistics (e.g., mean intensity, kurtosis) and texture features (e.g., uniformity and energy, contrast, IDM), were extracted using PyRadiomics (version 3.0.1). Intra-class correlation coefficient (ICC) was used to evaluate the consistency of feature extraction between two radiologists (ICC > 0.75 was considered high stability), and features with high stability were finally retained for model construction.

#### Outcome definition

The primary outcome event of this study was “confirmed TAK.” Referring to the 1990 ACR classification criteria and combining with the clinical practice of our center, TAK was defined as Takayasu’s Arteritis confirmed by the gold standard (8). Based on the above outcome definition, all patients included in the study were divided into two groups: TAK-confirmed group: Patients were confirmed to have TAK during the study period and met at least one of the following criteria: Met at least 3 of the 6 classification criteria for Takayasu’s Arteritis in the 1990 ACR criteria, and the first criterion must be included (9), Vascular biopsy confirmed the presence of typical chronic granulomatous inflammatory changes, Two senior rheumatologists unanimously determined TAK based on complete clinical data. Non-TAK group: Patients did not develop TAK during the entire study period and met all of the following conditions: Had a clear alternative diagnosis to explain their clinical manifestations, Did not show characteristic manifestations of TAK after at least 6 months of follow-up, TAK diagnosis was unanimously excluded by two senior rheumatologists after review [Note: The 6 classification criteria for Takayasu’s Arteritis in the 1990 ACR: (1) Age of onset ≤ 40 years, (2) Intermittent claudication of the extremities, (3) Weakened brachial artery pulse, (4) Difference in systolic blood pressure between the two upper extremities > 10 mmHg, (5) Vascular murmur, (6) Abnormal arteriography]. Early-stage TAK was defined as TAK with symptom duration ≤6 months from the onset of initial non-specific symptoms (e.g., fever, fatigue, joint pain) to confirmed diagnosis, without obvious vascular stenosis, occlusion, dilation, or aneurysm formation confirmed by CTA or angiography. Patients with late-stage vascular damage (e.g., established stenosis, occlusions, aneurysms) were actively excluded during patient screening to ensure the model targets early-stage diagnosis.

#### Statistical analysis

The sample size estimation in this study followed the principle of the number of events, the core of which was to ensure a sufficient number of outcome events to prevent model overfitting. According to the core standard, the event count for each variable required at least Events Per Variable (EPV) ≥ 5 (10). It was estimated that, based on preliminary data, previous literature reviews, and clinical experience, the estimated incidence of early-stage TAK in suspected cases was approximately 30% (11). This incidence was consistent with previous studies on TAK-diagnosed cohorts, indicating that the population included in this study had sufficient risk heterogeneity and clinical representativeness, and was suitable for the construction and validation of a prediction model for early-stage TAK in suspected cases. The minimum number of required events was calculated according to the principle of EPV ≥ 5: E (number of required events) = EPV × V = 5 × 8 = 40 events. Based on the estimated event incidence (*P* ≈ 30%), the total sample size required was estimated to be at least: 40 (events)/0.30 (incidence) ≈ 133 patients. Considering that there might be approximately 20% data missing or excluded during the study, the sample size was increased by 20%. The final target sample size was: 133/(1–0.30) ≈ 190 patients. The patient cohort included in this study (from January 2018 to December 2024) exceeded this limit. Setting the target sample size was to ensure sufficient statistical validity of this study. Statistical analysis was performed using SPSS 26.0 and R 4.2.3. First, in the training set, all variables were subjected to Logistic regression analysis to further determine the independent influencing factors for early-stage TAK diagnosis in suspected cases (*p* < 0.05), and their odds ratios (OR) and 95% confidence intervals (CI) were calculated. Based on the finally determined independent influencing factors, a nomogram model was constructed using the rms package. The receiver operating characteristic (ROC) curve was plotted, and the areas under the curve (AUC) value was calculated. A model was considered to have good accuracy when the AUC value was between 0.7 and 0.9, and extremely high accuracy when > 0.9. The calibration curve was plotted and evaluated using the Hosmer-Lemeshow goodness-of-fit test. The closer the calibration curve was to the 45-degree diagonal and the *p* value of the H-L test>0.05, the better the consistency between the model-predicted probability and the actual incidence. Decision curve analysis (DCA) was used to evaluate the clinical application value of the nomogram by calculating the net benefit at different threshold probabilities.

## Results

### Comparison of general data of patients in the training set and validation set

A total of 356 patients were included in this study. According to a 7:3 ratio, 356 patients were divided into a training dataset (249 cases) and a validation set (107 cases). Among the 249 patients in the training set, 75 cases (30.12%) were confirmed to have early-stage TAK in suspected cases, and 174 cases (69.88%) were non-TAK. Among the 107 patients in the validation set, 32 cases (29.91%) were confirmed to have early-stage TAK, and 75 cases (70.09%) were non-TAK. There were no statistically significant differences in the general data of patients between the training set and the validation set (*p* > 0.05) (Table 1). In the non-TAK group (*n* = 249), the final confirmed alternative diagnoses were as follows: atherosclerosis (*n* = 68, 27.31%), giant cell arteritis (*n* = 23, 9.24%), syphilitic aortitis (*n* = 15, 6.02%), arterial dissection (*n* = 12, 4.82%), fibromuscular dysplasia (*n* = 11, 4.42%), other autoimmune diseases (e.g., systemic lupus erythematosus, *n* = 9, 3.61%), and other conditions (e.g., vascular trauma, *n* = 111, 44.58%). In the TAK group (*n* = 107), the distribution of vascular lesions was as follows: aortic arch (62.67%), brachiocephalic trunk (54.67%), subclavian artery (48.00%), common carotid artery (42.67%), thoracic aorta (33.64%), abdominal aorta (28.97%), and renal artery (18.69%).

**Table 1:** Comparison of general data of patients in the training set and validation set.

Indicators		Training set (*n* = 249)	Validation set (*n* = 107)	*t/χ* ^2^	*P*
Age		41.19 ± 14.91	42.27 ± 14.60	0.631	0.529
Gender	Male	49 (19.68)	22 (20.56)	0.037	0.849
	Female	200 (80.32)	85 (79.44)		
BMI (kg/m^2^)		22.43 ± 3.35	22.79 ± 3.01	0.917	0.360
Disease duration (months)		18.53 ± 9.31	17.92 ± 8.70	0.578	0.564
Unexplained fever	Yes	70 (28.11)	28 (26.17)	0.142	0.707
	No	179 (71.89)	79 (73.83)		
Fatigue or weight loss	Yes	132 (53.01)	54 (50.47)	0.194	0.659
	No	117 (46.99)	53 (49.53)		
Intermittent claudication of limbs	Yes	108 (43.37)	43 (40.19)	0.311	0.577
	No	141 (56.63)	64 (59.81)		
Vascular murmur	Yes	141 (56.63)	57 (53.27)	0.341	0.559
	No	108 (43.37)	50 (46.73)		
Erythrocyte sedimentation rate (ESR, mm/h)		48.63 ± 22.27	46.91 ± 21.80	0.672	0.502
C-reactive protein (CRP, mg/L, M)		26.31 ± 22.42	24.19 ± 20.76	0.836	0.404
White blood cell count (×10^9^/L)		7.81 ± 2.64	7.70 ± 2.54	0.365	0.716
Platelet count (×10^9^/L, M)		301.26 ± 86.14	291.98 ± 82.96	0.942	0.347
Thickest part of the vascular wall (mm)		4.21 ± 1.37	4.09 ± 1.45	0.745	0.457
Degree of vascular wall enhancement	Strong	187 (75.10)	78 (72.90)	0.191	0.662
	Weak	62 (24.90)	29 (27.10)		
Mean intensity		86.42 ± 24.71	84.91 ± 25.37	0.524	0.600
Kurtosis		3.80 ± 1.19	3.72 ± 1.33	0.561	0.575
Uniformity and energy (Texture features reflecting the homogeneity of vascular wall imaging signals)		0.42 ± 0.15	0.44 ± 0.16	1.130	0.259
Homogeneity/IDM		0.38 ± 0.12	0.39 ± 0.13	0.703	0.483
Contrast		1.85 ± 0.76	1.79 ± 0.81	0.670	0.504
SRE		0.64 ± 0.21	0.62 ± 0.23	0.800	0.424
LRE		1.32 ± 0.45	1.35 ± 0.47	0.569	0.570
Zone variance		285.62 ± 89.27	278.38 ± 92.10	0.695	0.488

BMI, body mass index; ESR, erythrocyte sedimentation rate; CRP, C-reactive protein; IDM, Inverse Difference Moment (a texture feature); SRE, Short-Run Emphasis; LRE, Long-Run Emphasis. Radiomics features were extracted from the arterial wall region of interest on CTA.

### Univariate analysis of influencing factors for early-diagnosed Takayasu arteritis patients

Univariate analysis showed that in the training set, among the suspected cases, there were statistically significant differences (*p* < 0.05) in the indicators of intermittent claudication of limbs, vascular murmur, ESR, CRP, the thickest part of the vascular wall, the degree of vascular wall enhancement, uniformity and energy, and contrast between the early-diagnosed TAK patients and non-TAK patients (Table 2).

**Table 2:** Univariate analysis of influencing factors for early diagnosis of whether a suspected case is a TAK patient.

Indicators		Diagnosed group (*n* = 75)	Undiagnosed group (*n* = 174)	*t/χ* ^2^	*P*
Age		38.56 ± 15.24	41.62 ± 14.73	1.488	0.138
Gender (*n*, %)	Male	12 (16)	37 (21.26)	0.919	0.338
	Female	63 (84)	137 (78.74)		
BMI (kg/m^2^)		22.23 ± 3.61	22.79 ± 3.22	1.213	0.226
Disease duration (months)		19.02 ± 9.06	17.99 ± 8.54	0.857	0.392
Unexplained fever (*n*, %)	Yes	25 (33.33)	45 (25.86)	1.448	0.229
	No	50 (66.67)	129 (74.14)		
Fatigue or weight loss (*n*, %)	Yes	45 (60)	87 (50)	2.104	0.147
	No	30 (40)	87 (50)		
Intermittent claudication of limbs (*n*, %)	Yes	42 (56)	66 (37.93)	6.967	0.008
	No	33 (44)	108 (62.07)		
Vascular murmur (*n*, %)	Yes	54 (72)	87 (50)	10.328	0.001
	No	21 (28)	87 (50)		
Erythrocyte sedimentation rate (ESR, mm/h)		56.76 ± 19.24	49.92 ± 12.34	3.357	0.001
C-reactive protein (CRP, mg/L, M)		42.31 ± 23.45	32.69 ± 18.76	3.434	0.001
White blood cell count (×10^9^/L)		8.81 ± 2.64	8.19 ± 2.34	1.844	0.066
Platelet count (×10^9^/L, M)		338.26 ± 86.14	316.98 ± 79.96	1.882	0.061
Thickest part of the vascular wall (mm)		4.72 ± 1.33	4.13 ± 1.16	3.520	0.001
Degree of vascular wall enhancement (*n*, %)	Strong	65 (86.67)	122 (70.11)	7.678	0.006
	Weak	10 (13.33)	52 (29.89)		
Mean intensity		87.93 ± 25.86	84.23 ± 22.51	1.137	0.257
Kurtosis		3.92 ± 1.41	3.62 ± 1.10	1.808	0.072
Uniformity and energy		0.36 ± 0.15	0.41 ± 0.13	2.592	0.010
Homogeneity/IDM		0.38 ± 0.12	0.41 ± 0.11	1.921	0.056
Contrast		2.42 ± 0.85	2.17 ± 0.52	2.841	0.005
SRE		0.66 ± 0.22	0.68 ± 0.20	0.702	0.483
LRE		1.34 ± 0.45	1.28 ± 0.42	1.012	0.313
Zone variance		278.31 ± 90.13	265.82 ± 85.27	1.042	0.298

### Multivariate logistic regression analysis of influencing factors for early-diagnosed TAK patients among suspected cases

Taking the differences in the influencing factors of TAK patients in early diagnosis as the dependent variable (undiagnosed group = 0, diagnosed group = 1), and taking intermittent claudication of limbs, vascular murmur, ESR, CRP, the thickest part of the vascular wall, the degree of vascular wall enhancement, uniformity and energy, and contrast (*p* < 0.05) as covariates, a further multivariate Logistic regression analysis was carried out (Supplementary Table S1). The results showed that intermittent claudication of the limbs, vascular murmur, ESR, CRP, the thickest part of the vascular wall, degree of vascular wall enhancement, and contrast were independent risk factors for early-stage TAK (all *p* < 0.05). An OR value >1 indicates that the presence or higher values of these features are associated with an increased risk of confirmed TAK. The OR for uniformity and energy was <1 (0.632), indicating that this radiomics feature serves as a protective factor, and its higher values are associated with a reduced risk of developing TAK (*p* < 0.05) (Table 3).

**Table 3:** Multivariate logistic regression analysis of early-diagnosed TAK patients among suspected cases.

Indicators	β	SE	Wald	*P*	OR	95%CI
Intermittent claudication of limbs	0.512	0.245	4.365	0.037	1.669	1.032–2.698
Vascular murmur	0.687	0.312	4.847	0.028	1.987	1.077–3.666
ESR	0.031	0.010	9.610	0.002	1.032	1.011–1.053
CRP	0.025	0.007	12.755	0.001	1.025	1.011–1.040
The thickest part of the vascular wall	0.405	0.118	11.789	0.001	1.499	1.189–1.890
Degree of vascular wall enhancement	0.632	0.301	4.414	0.036	1.882	1.043–3.397
Uniformity and energy	−1.102	0.527	4.371	0.017	0.632	0.418–0.832
Contrast	0.698	0.220	10.074	0.002	2.010	1.306–3.092

### Establishment of the nomogram prediction model

**Figure 1:**
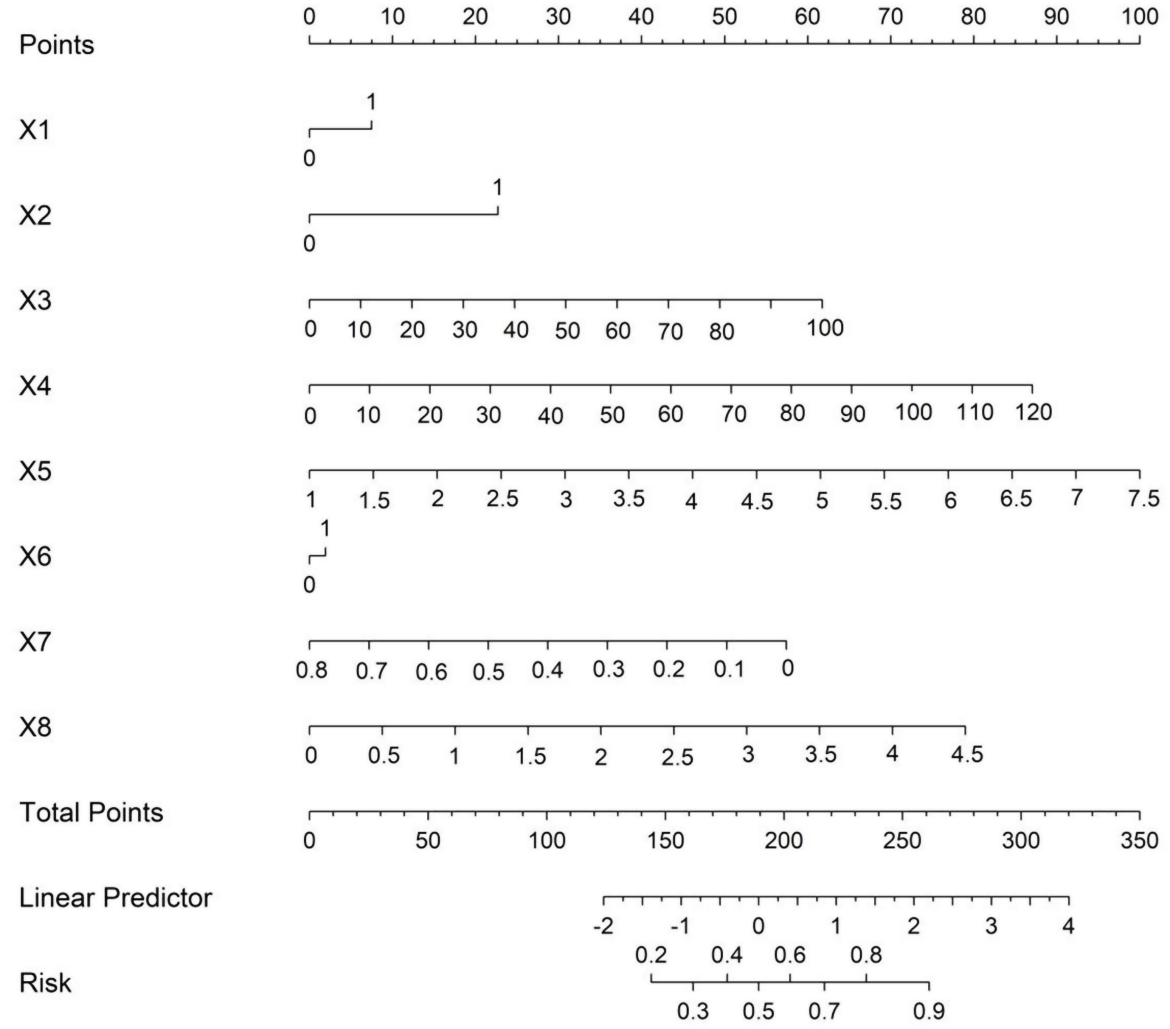
Nomogram prediction model for the risk of early-diagnosed TAK patients among suspected cases.

Based on the results obtained from the multivariate Logistic regression analysis, a nomogram prediction model for the influencing factors of early-diagnosed TAK patients was constructed. In the constructed nomogram, each risk factor was assigned a specific scale segment. By accurately locating the actual values of each risk factor of the patient on the corresponding scale segment and then projecting vertically upward, the score values corresponding to each risk factor could be obtained. By summing up these score values, the total score was obtained. The predicted probability value corresponding to the total score represented whether the patient was diagnosed with TAK in early diagnosis (Figure 1).

### Evaluation and validation of the nomogram prediction model

**Figure 2:**
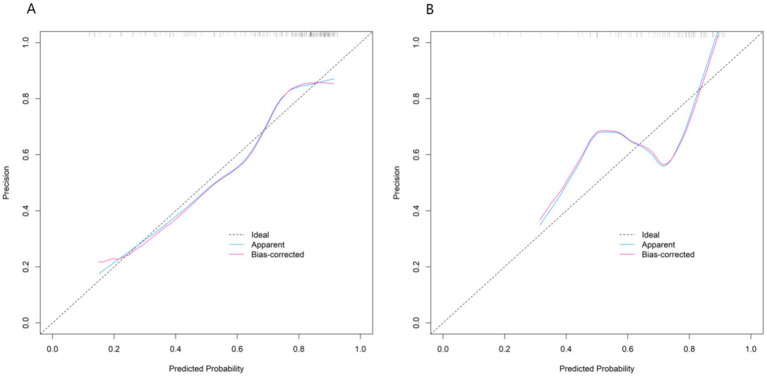
Calibration curves for early-diagnosed TAK patients among suspected cases (**A**: training set, **B**: validation set).

**Figure 3:**
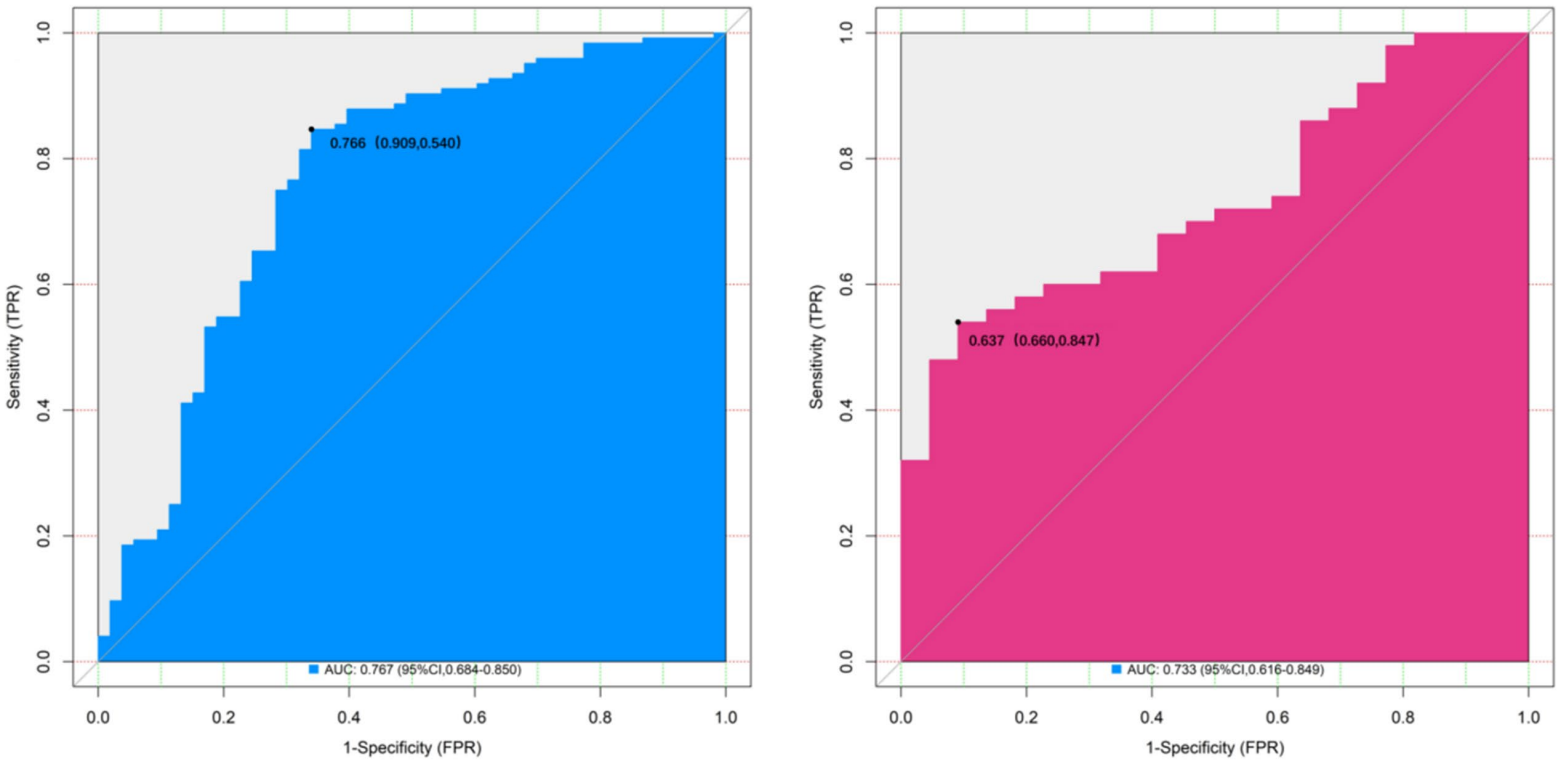
ROC curves for early-diagnosed TAK patients among suspected cases (**A**: training set, **B**: validation set).

The nomogram model showed good calibration and goodness-of-fit between the predicted values and the actual values in both the training set and the validation set (the C-index was 0.767 and 0.733 respectively, the mean absolute errors of the conformity between the predicted values and the true values were 0.163 and 0.180 respectively, and the results of the Hosmer-Lemeshow test were χ^2^ = 7.937, *p* = 0.440 and χ^2^ = 11.924, *p* = 0.155 respectively). The ROC curve showed that the AUC of the nomogram model predicting whether the patient was diagnosed with TAK in early diagnosis in the training set and the validation set were 0.767 (95% CI: 0.684–0.850) and 0.733 (95% CI: 0.616–0.849) respectively, and the sensitivities and specificities were 0.847, 0.660 and 0.720, 0.500, respectively. The results showed that the model exhibited high prediction performance in both the training dataset and the validation dataset. The calibration curve is shown in Figure 2, and the ROC curve is shown in Figure 3. To evaluate the incremental value of the proposed nomogram, we compared its diagnostic performance with the 1990 ACR classification criteria in the same cohort. The results showed that in the training set, the nomogram had an AUC of 0.767 (95% CI: 0.684–0.850), sensitivity of 0.847, and specificity of 0.660, while the 1990 ACR criteria had an AUC of 0.689 (95% CI: 0.598–0.780), sensitivity of 0.720, and specificity of 0.683. In the validation set, the nomogram had an AUC of 0.733 (95% CI: 0.616–0.849), sensitivity of 0.720, and specificity of 0.500, compared with the 1990 ACR criteria (AUC: 0.652, 95% CI: 0.528–0.776; sensitivity: 0.625; specificity: 0.533).

### Decision curve analysis of the nomogram prediction model

**Figure 4:**
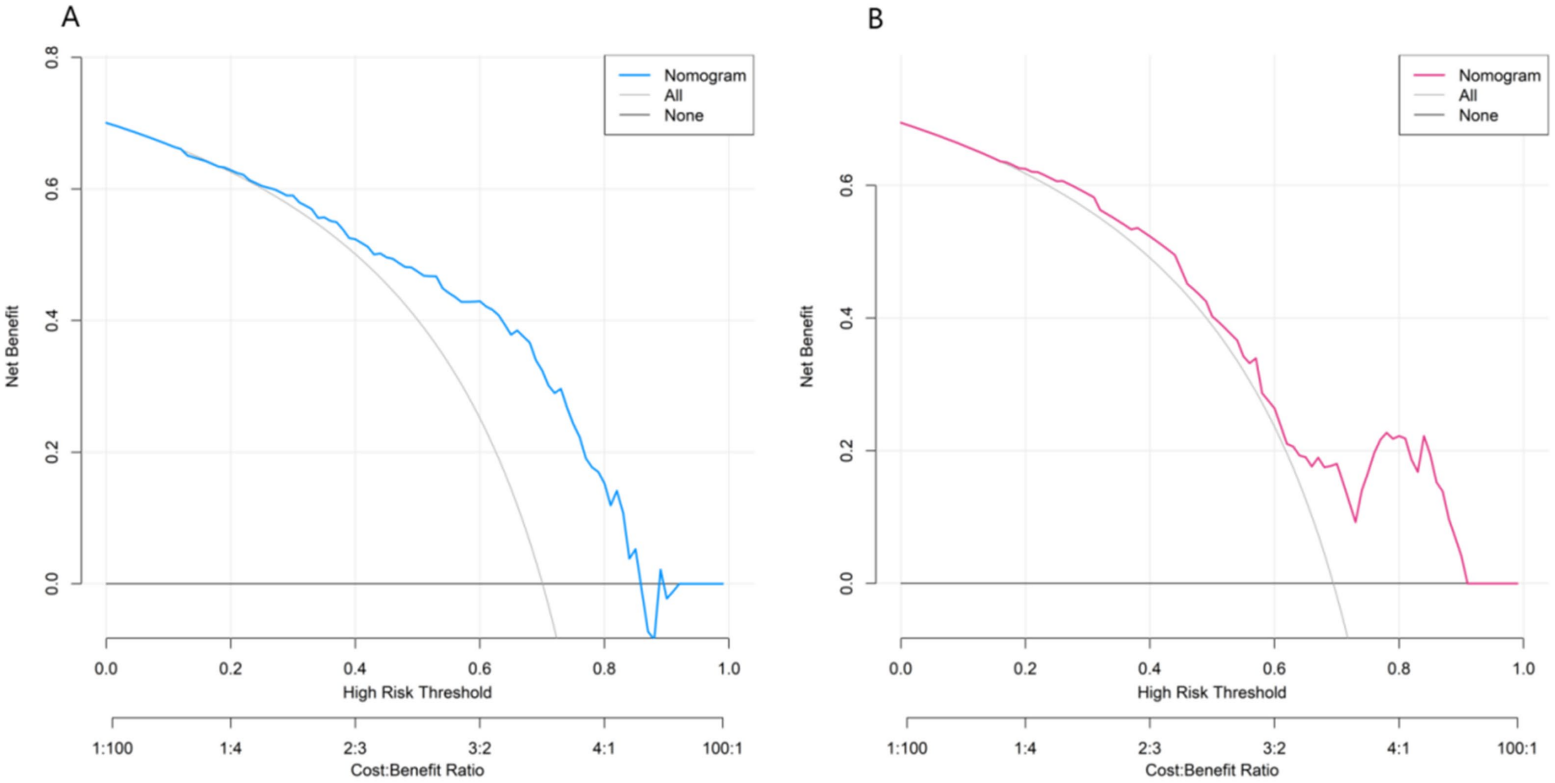
Decision curves for early-diagnosed TAK patients among suspected cases (**A**: Training set, **B**: Validation set).

The decision curve showed that when the threshold probability was approximately between 0.1 and 0.9, the decision of using the nomogram model constructed in this study to predict the risk differences caused by the influencing factors of whether the patient was diagnosed with TAK in early diagnosis had more clinical benefits than the decision of considering all patients to be damaged or all patients to be undamaged before surgery (Figure 4).

## Discussion

Based on the clinical practice in a general hospital, a clinical-radiomics nomogram model integrating eight predictors was successfully developed and validated in this study. The model demonstrated excellent diagnostic performance in both the training set and the validation set, with AUC values reaching 0.767 (95%CI: 0.684–0.850) and 0.733 (95%CI: 0.616–0.849) respectively, indicating its good discriminatory ability. The calibration curve showed a good agreement between the predicted probability and the actual observed probability, and the decision curve analysis further confirmed that the model had significant clinical net benefits within a wide range of threshold probabilities.

Through systematic univariate analysis, eight statistically significant predictors were screened from multiple candidate indicators in this study. These factors reflect the pathophysiological process of TAK from different aspects (12, 13). In terms of clinical manifestations, intermittent claudication of the limbs and vascular murmurs are direct manifestations of hemodynamic changes caused by vascular stenosis. Although these two signs are relatively subjective, they are of great value in clinical practice due to their easy acquisition (14, 15). In laboratory examinations, ESR and CRP, as acute-phase reaction proteins, reflect the systemic inflammatory load, which is an important biological marker of TAK disease activity. In conventional imaging, the thickest part of the vascular wall and the degree of vascular wall enhancement are direct evidence of local active inflammation in the vascular wall. These features can be accurately identified on CTA images and have high diagnostic specificity (16, 17).

The most innovative finding of this study lies in the incorporation of radiomics features into the diagnostic model. The two texture features, uniformity and energy and contrast, showed significant differences between the confirmed group and the unconfirmed group, indicating characteristic changes in the microstructure of the diseased vascular wall in TAK. uniformity and energy reflects the uniformity of the image texture. A decrease in its value may suggest an increase in the heterogeneity of the vascular wall tissue structure. Contrast reflects the intensity difference between adjacent pixels. An increase in its value may reflect changes in tissue components caused by inflammation. These radiomics features can quantify the texture information that cannot be recognized by the human eye, providing new biological evidence for early diagnosis (18, 19).

From a pathophysiological perspective, the eight predictors included in this study actually reflect different stages of the occurrence and development of TAK (20, 21). In the early stage of the disease, changes in the microstructure of the vascular wall may be reflected by radiomics features. Subsequently, the systemic inflammatory response is reflected by ESR and CRP indicators (22). When the inflammation progresses to the full-thickness of the vascular wall, it is manifested as vascular wall thickening and enhancement on CTA. As vascular stenosis forms, clinical manifestations such as intermittent claudication of the limbs and vascular murmurs gradually appear. Therefore, our model actually covers the complete pathological process from the molecular level, histology to hemodynamic changes, which may be the underlying reason for its superior diagnostic performance (23, 24).

Compared with the existing literature (25), this model shows obvious advantages. Previous studies mainly focused on the application of single-type indicators. This study significantly improved the diagnostic efficacy by integrating information from four dimensions: clinical, laboratory, conventional imaging, and radiomics. In particular, the addition of radiomics features brought incremental information that traditional methods could not provide, which may be the key factor for the performance improvement.

The clinical practical value of this study is manifested in multiple aspects. First, the nomogram presents a complex mathematical model in an intuitive graphical way. Clinicians only need to locate the scores on the eight corresponding axes according to the specific conditions of patients, and then obtain the individualized probability of disease after summarizing, which is easy to operate and popularize. Second, the decision curve analysis shows that using this model can obtain clinical net benefits within a wide range of threshold probabilities, ensuring its effectiveness in practical application. In addition, this model can also be used for risk stratification of patients, helping doctors identify high-risk patients who need active intervention and optimizing the allocation of medical resources. Compared with the 1990 ACR criteria, the proposed nomogram showed superior diagnostic performance, especially in sensitivity (0.847 vs. 0.720 in the training set and 0.720 vs. 0.625 in the validation set). This advantage is particularly important for early TAK, as the 1990 ACR criteria are more suitable for typical late-stage cases and have limited sensitivity for early-stage patients. The integration of radiomics features and multi-dimensional indicators enables the nomogram to capture subtle changes in the early stage of TAK, thereby improving diagnostic sensitivity while maintaining comparable specificity. This confirms the clinical value of the nomogram as a supplement to existing diagnostic tools (26).

However, the limitations of this study must be objectively recognized. First, this is a single-center retrospective study. Although strict internal validation was carried out, selection bias is difficult to completely avoid. Before the model is widely promoted, external validation in a multi-center, prospective cohort is required. Second, the reproducibility of radiomics analysis is affected by multiple factors, including CT scanning parameters, contrast agent injection protocols, etc. Although these effects were minimized through standardized procedures, the applicability among different medical institutions still needs further verification (27). Third, although manual segmentation of the region of interest ensures accuracy, it is time-consuming. Developing an automatic segmentation algorithm in the future will greatly improve the efficiency of this method.

Based on the findings and limitations of this study, the following future research directions are proposed: First, conduct multi-center prospective studies to verify the generalization ability of the model, second, explore the association between radiomics features and disease activity and prognosis, and construct a model for efficacy evaluation and prognosis prediction, third, apply more advanced algorithms such as deep learning to feature extraction and model construction, fourth, combine multi-omics data such as genomics and proteomics to construct a more comprehensive diagnostic system.

In conclusion, a clinical-radiomics nomogram model containing eight predictors was developed and validated in this study. This model integrates clinical manifestations, laboratory examinations, conventional imaging features, and radiomics features, providing a powerful tool for the early diagnosis of TAK (14, 28). Through the integration of multi-dimensional information, this model not only significantly improves the diagnostic accuracy but also has good clinical practicability and interpretability, and is expected to play an important role in clinical practice.

In summary, this study developed and validated a clinical-radiomics nomogram integrating intermittent claudication of the limbs, vascular murmurs, ESR, CRP, the thickest part of the vascular wall, the degree of vascular wall enhancement, uniformity and energy, and contrast. This model can provide individualized and quantitative risk assessment for the early diagnosis of TAK, helping clinicians make accurate risk stratification and early intervention decisions for suspected patients.

## Data Availability

The original contributions presented in the study are included in the article/Supplementary material, further inquiries can be directed to the corresponding author.
