# Supplementary material for: Construction and internal cohort verification of clinical-imaging-based nomogram for early diagnosis in Takayasu arteritis
Source: Front Med (Lausanne). 2026 Feb 3;13:1743349. doi: 10.3389/fmed.2026.1743349 (PMC12960647; doi:10.3389/fmed.2026.1743349)
Supplement: Supplementary file 1 [file Table_1.docx]

**Supplemental Table 1.** Variable assignment table

| Variable | Meaning | Assignment |
| --- | --- | --- |
| X1 | Intermittent claudication of limbs | Yes=0, No=1 |
| X2 | Vascular murmur | Yes=0, No=1 |
| X3 | ESR | Continuous variable |
| X4 | CRP | Continuous variable |
| X5 | The thickest part of the vascular wall | Continuous variable |
| X6 | Degree of vascular wall enhancement | Strong=0, Weak=1 |
| X7 | uniformity and energy | Continuous variable |
| X8 | Contrast | Continuous variable |
| Y | Whether a patient is a TAK patient in early diagnosis | Diagnosed group=0, Undiagnosed group=1 |
